# Cross-context Validation of a Technostress Scale for the Aging Workforce

**DOI:** 10.1097/JOM.0000000000003349

**Published:** 2025-02-20

**Authors:** Anna Comotti, Alice Fattori, Cristina Di Tecco, Pasquale Bufano, Marco Laurino, Francesca Mastorci, Simone Russo, Teresa Barnini, Luca Ferrari, Catalina Ciocan, Matteo Bonzini

**Affiliations:** From the Occupational Medicine Unit, Foundation IRCCS Ca’ Granda Ospedale Maggiore Policlinico, Milan, Italy (A.C., T.B., M.B.); Department of Clinical Science and Community Health, University of Milan, Milan, Italy (A.F., L.F., M.B.); Department of Occupational and Environmental Medicine, Epidemiology and Hygiene, INAIL, Monte Porzio Catone, Rome, Italy (C.D.T., S.R.); Institute of Clinical Physiology, National Research Council, Pisa, Italy (P.B., M.L., F.M.); Department of Psychology, University of Milano-Bicocca, Milan, Italy (T.B.); and Department of Public Health and Pediatrics, University of Turin, Turin, Italy (C.C.).

**Keywords:** technostress, validation, older workers, workability, aging

## Abstract

**Objective:**

This study examines the psychometric properties of a workplace-adapted version of an existing Technostress scale to assess technostress in workers over 50 across different companies.

**Methods:**

Data were collected from 470 workers over 50 across finance, packaging, and steel sectors. We evaluated the internal consistency, criterion validity, and factor structure of the Technostress scale using Cronbach’s alpha, McDonald’s omega, confirmatory factor analysis, and correlations with stress, coping, well-being, and workability.

**Results:**

Scale and subscales demonstrated strong reliability (coefficients > 0.75) and validity. Confirmatory factor analysis confirmed bifactor model with optimal fit indices (comparative fit index = 0.98, Tucker-Lewis Index = 0.96, root mean square error of approximation = 0.05). Technostress correlated positively with stress and negatively with well-being and workability. Significant differences in technostress were observed by sex and occupational role.

**Conclusions:**

The adapted scale is a reliable tool for evaluating technostress among older workers, supporting tailored interventions to enhance workplace well-being and productivity.

## Abstract 2

This study provides a validated tool to assess technostress among older workers, facilitating early identification of tech-related stressors. Clinicians and occupational health professionals can use these insights to implement tailored interventions, favoring workplace well-being, productivity, and retention of aging employees in technology-driven environments.

## Graphical abstract

**Figure:**
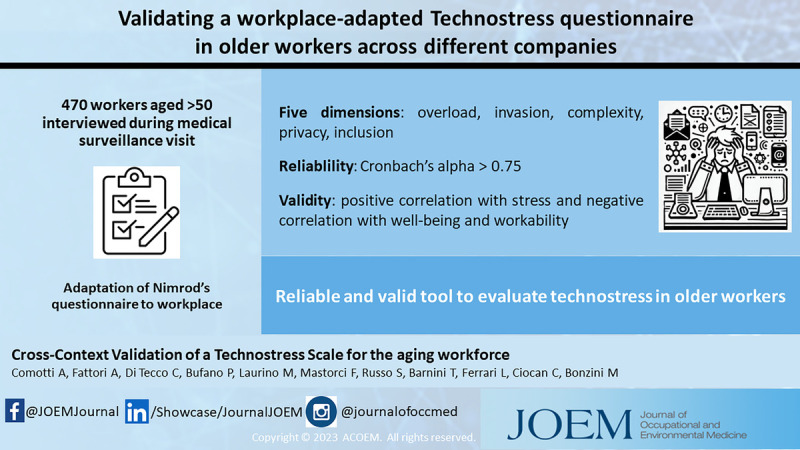

**LEARNING OUTCOMES**

By the end of this article, readers will be able to:

- Identify the key dimensions of technostress (overload, invasion, complexity, privacy, and inclusion) affecting workers over 50 and understand how these dimensions may vary.
- Evaluate the psychometric properties of the workplace-adapted Technostress scale, including its reliability, criterion validity, and factor structure.
- Understand the importance of targeted interventions to improve well-being and productivity in technology-integrated workplaces.

In the contemporary and rapidly evolving technological advancements, the integration of technology in professional environments has become widespread, transforming the way individuals engage with their work tasks.^1,2^ While technological innovations offer numerous benefits, the pervasiveness of technology in the workplace has also given rise to a combination of negative outcomes.^3^ Specifically, new evidence has conceptualized a novel phenomenon known as technostress, which pertains to the strain process triggered by the use of information and communication technology (ICT).^4^ Since its first definition by Brod in 1984,^5^ who described technostress as “*a modern disease of adaptation caused by the inability to adapt or cope with new ICTs in a healthy manne*r,” many researchers over the years have formulated and expanded the definition of technostress, mostly focusing on the information overload and on the constant availability demanded by ICT, both in the workplace and in private life.^6^ One of the most cited definition considers technostress as the stress associated with the pervasive use of ICT, resulting from “*the fallouts of an individual’s attempts and struggles to deal with constantly evolving ICTs and the changing cognitive and social requirements related to their use.*”^7^

In occupational settings, different stressors can contribute to generating technostress.^8,9^ Research has primarily highlighted how the constant connectivity and availability expectation from supervisor and colleagues induce workers to be constantly available to receive work requests and to use work-related ICT during nonworking time (namely techno-invasion), also posing a threat to the work-life balance.^10,11^

Another commonly recognized risk factor for technostress is communication information overload (ie, techno-overload) which refers to the challenging circumstances of managing a higher volume of information, rapidly delivered through technology and frequently received from several simultaneous channels, for which it is difficult to establish priorities. An additional contributing factor to technostress is techno-complexity, the perception that ICTs require too much efforts in relation to employee’s skills, which is often associated to feelings of inadequacy.^7,12^ The larger integration of technology in the workplace has also raised concerns about the potential impact on the aging workforce. Indeed, of particular interest in recent research is the observation that technostress might not be uniformly distributed across all demographic groups, with evidence suggesting that older adults could be differently challenged by techno-stressors.^13,14^ Research on occupational technostress, although limited, indicates that workers aged over 50 could be particularly triggered by the constant changes occurring in ICT and may experience high levels of techno-complexity, techno-invasion, and techno-uncertainty.^15^ Compared to younger colleagues, older workers could also experience a sense of inferiority^13^ and lower levels of computer self-efficacy, which in turn can increase the association between technology-mediated interruptions and stress.^12^

Moreover, psychosocial risks, such as poor work organization, lack of effective management in the use of technologies, inadequate social support, and request of constant availability, can exacerbate technostress, particularly among older workers who may also face digital literacy. Limited digital skills can increase techno-complexity and uncertainty, further affecting their ability to adapt to technological demands.^16^ As organizations increasingly embrace digital innovations to enhance productivity and streamline operations and the proportion of older workers continues to grow,^17^ it becomes imperative to understand the complexities surrounding age-related variations in technostress by adopting suitable psychometric tools. Older workers typically bring significant experience and knowledge to the workplace. Supporting them in facing digital transformations can enhance their well-being, productivity, and job satisfaction.^18^

In this respect, a new scale specifically designed to measure technostress among older people was recently developed by Nimrod.^13^ The questionnaire draws on relevant findings from technostress literature^3,7,19^ also adding a specific threat as techno-inclusion, which refers to a feeling of inadequacy in comparison to younger users, resulting in a sense of pressure to be included in the modern tech environment.^20^ The scale proved to be a reliable measurement to investigate technostress among older adults; however, it focused on features experienced in later life with no specific reference to the occupational dimension. Since no psychometric tools are currently available to specifically assess work-related technostress among older workers, we aim to adapt Nimrod’s scale to workplace settings by testing the questionnaire among an aging Italian workforce from different occupational sectors. We selected companies from the finance, packaging, and steel industries, representing a broader range of technologies and work demands, to capture different contexts and ensure the scale is tested across various environments. We also aim to assess the scale’s associations with occupational variables and to identify potential intervention strategies, specifically targeted for older workers facing technological tools and changes.

## METHODS

### Participants

Data were acquired as part of the PROAGEING project, whose protocol was published elsewhere.^21^ All full-time workers, aged over 50 in the selected industries were invited to participate on a voluntary basis. Interviews were conducted by occupational physicians during the medical surveillance required by Italian Legislation (Legislative Decree 81/08). This approach ensured a high participation rate (>60%,^22^). Research Electronic Data Capture^23^ was used for data collection, ensuring that all fields were completed before submission, so no missing data were recorded.

A list of questionnaires including the Technostress scale,^13^ the Perceived Stress Scale (PSS^24^), the World Health Organization Well-Being Index (WHO-5^25^), the Work Ability Index (WAI^26^), the Coping orientation to problems Experienced^27^ was administered to the participants.

A total of 470 workers completed the questionnaires, with median age of 55 years (interquartile range = 52–58). Participants were predominately males (*n* = 354; 76%) with 231 (49%) working in bank, 134 (29%) in a packaging industry, and 103 (22%) in a steel industry. Subjects were categorized as either white-collar workers (*n* = 289, 62%) and blue-collars workers (*n* = 179, 38%) based on their occupational sector. A full description of sample’s characteristics was previously published.^22^

### Questionnaire

The Italian version of the Technostress scale, originally developed by Nimrod and specifically designed for older adults, was administered to a population of workers aged 50 and above.^13^ The questionnaire has been readapted for the workplace setting, meaning that every question specifically referenced technologies at work rather than technologies in general. For instance, the first item “*This technology makes me do thing slower*” has been modified into “*This use of technologies at work makes me do thing slower.*”

The questionnaire consisted of 14 items related to five dimensions: overload, invasion, complexity, privacy, and inclusion. Overload refers to the burden of coping with excessive problems, resulting in slower task performance. Invasion denotes how technology permeates into daily life, mixing public and personal areas. Complexity deals with the challenges of constantly changing technology, which can be hard to understand and adjust to. Privacy concerns potential risks associated with personal information being tracked through ICT usage. Inclusion captures the sense of inferiority experienced by older users compared to younger counterparts.

Participants responded using a five-point Likert scale, ranging from 1 (strongly disagree) to 5 (strongly agree). Five scores were computed for each dimension by averaging the corresponding item scores, and a total Technostress score was derived by summing up these five subscores.

The Technostress scale was originally developed in English. To ensure cross-cultural validity, experts in English language conducted a direct translation of the scale into Italian. Occupational psychologist experienced in working with aging employees reviewed the translation. Physicians who administered the scale during structured interviews reported no issues with participant comprehension.

### Statistical Analysis

The internal consistency of the questionnaire and its subscales was assessed using Cronbach’s alpha and McDonald’s omega coefficients. A value exceeding 0.70 was considered indicative of satisfactory reliability. To investigate criterion validity, we evaluated the concurrent validity through the associations between technostress and health outcome variables. Coherently to previous findings, we hypothesized that technostress was positively associated to perceived stress and negatively associated to well-being and workability.^3,8^ In addition, we hypothesized that technostress was associated to coping strategies.^28^ Pearson’s correlation coefficients were calculated to examine the strength and direction of these relationships. Known-group validity explored potential differences among subgroups, known to differ on technostress. Because previous research showed that technostress was associated with sex, and educational level,^29^ we examined differences in technostress scores by sex, education, and occupational groups (ie, white-collars and blue-collars). These differences were analyzed using Student’s *t* test or one-way analysis of variance as appropriate. Before conducting the analyses, we verified the assumptions of normality (qq-plot showed that data not far from a normal distribution) and homogeneity of variances (Bartlett’s test, *P* > 0.05 for occupation, sex, and educational level). We estimated the effect size using Cohen’s d and Cohen’s f for comparisons between two and three groups mean differences respectively.

We tested the proposed five-factor structure of the tecnhostress scale using confirmatory factor analysis (CFA) using maximum likelihood estimator. Three models were compared: the unidimensional model, the traditional five-dimensional structure comprising overload, invasion, complexity, privacy, and inclusion dimensions and a bifactor model incorporating a general factor along with the five specific dimensions. Model fit indices, including comparative fit index (CFI), Tucker-Lewis Index (TLI), and root mean square error of approximation (RMSEA), were used to evaluate the goodness of fit. Adequate fit was considered when CFI and TLI values exceeded 0.90, and RMSEA values were below 0.05. Statistical significance was set at *P* < 0.05, and all analyses were conducted using R software (R Core Team, 2023; RFoundation for Statistical Computing, Vienna, Austria).

## RESULTS

Participants’ overall technostress ranged between 7 and 20, with mean value equal to 12.9 (SD = 2.1). Among the five dimensions, inclusion subscale reported the highest scores (3.4 ± 0.7). Scores exhibited a relatively normal distribution, with skewness of 0.3 and kurtosis of 3.5. Both Cronbach’s alpha and McDonald’s omega values for the overall scale (alpha: 0.75, 95%CI = 0.71–0.79, omega: 0.76, 95%CI = 0.69–0.80) as well as for its individual subscales (overload: alpha = 0.80, omega = 0.80; invasion: alpha = 0.74, omega = 0.75; complexity: alpha = 0.76, omega = 0.76; privacy: alpha = 0.81, omega = 0.85; inclusion: alpha = 0.62, omega = 0.64) indicated good reliability (Table 1).

**TABLE 1:** Summary Statistics: Mean (SD), Cronbach’s Alpha, and McDonald’s Omega of Scale and Subscales

	Mean (SD)	Cronbach’s Alpha (95% CI)	McDonald’s Omega (95% CI)
Overall technostress	12.7 (2.1)	0.75 (0.71, 0.79)	0.76 (0.69, 0.80)
Overload	2.3 (0.7)	0.80 (0.74, 0.84)	0.80 (0.61, 0.83)
Invasion	2.5 (0.8)	0.74 (0.68, 0.80)	0.75 (0.51, 0.74)
Complexity	2.5 (0.7)	0.76 (0.71, 0.80)	0.76 (0.59, 0.73)
Privacy	2.3 (0.7)	0.81 (0.76, 0.85)	0.85 (0.82, 0.91)
Inclusion	3.3 (0.7)	0.62 (0.56, 0.68)	0.64 (0.58, 0.71)

CFA indices for the three tested models are shown in Table 2: the unidimensional model, the five-dimensional structure and the bifactor model incorporating a general factor along with the five dimensions. While the unidimensional model reached the worse fit to the data, the 5-dimensional model demonstrated acceptable fit to the data, with CFI = 0.94, TLI = 0.92, RMSEA = 0.06, and SRMR = 0.05. In contrast, the bifactor model exhibited superior fit indices, with CFI = 0.98, TLI = 0.96, RMSEA = 0.05, and SRMR = 0.03, offering a better representation of the underlying structure, capturing both the shared variance among all items (general factor) and the unique variance specific to each dimension.

**TABLE 2:** CFA Results

	χ**^2^** (df)	df	CFI	TLI	SRMR	RMSEA (90% CI)
**Unidimensional**	1371.83	77	0.44	0.33	0.15	0.19 (0.18–0.20)
**5-Factor model**	197.046	67	0.94	0.92	0.06	0.06 (0.05–0.08)
**Bifactor model**	93.823	48	0.98	0.96	0.03	0.04 (0.03–0.06)

Three models were compared (unidimensional model including one general factor; 5-factors model including the five dimension of technostress; bifactor model including one general factor and the five dimensions of TS).

CFI, comparative fit index; RMSEA, root mean square error of approximation; SRMR, Standardized Root Mean Square Residual; TLI, Tucker-Lewis Index.

Technostress positively and significantly correlated with well-being impairment (WHO-5, r = 0.17, *P* < 0.001), perceived stress (PSS; r = 0.32, *P* < 0.001), avoidant coping subscale (r = 0.20, *P* < 0.001), and negatively correlated with workability (WAI; r = −0.24, *P* < 0.001). No significant correlation occurred with problem focused and emotional focused coping strategies.

We observed significant differences by sex (*P* = 0.007) with higher scores among females (mean = 13.3, SD = 2.04) compared to males (mean = 12.7, SD = 2.05), especially in the invasion (2.8 vs 2.5) and the inclusion (3.4 vs 3.2) dimensions. Overall, considering the whole scale, white-collars workers reported lower level of technostress compared to blue-collar workers (12.7 vs 13.1, *P* = 0.02). Considering the five individual subscales, white collars have shown higher scores in invasion (2.7 vs 2.3, *P* < 0.001) and privacy (2.3 vs 2.2, *P* = 0.04), while blue collars reported higher scores in overload (2.6 vs 2.1, *P* < 0.001), complexity (2.6 vs 2.4, *P* = 0.001), and inclusion (3.4 vs 3.2, *P* = 0.002) dimensions. The highest effect size was observed for overload and invasion (Table 3). We obtained similar results and trends considering educational level, as it reflects and overlaps with job type (Table 3).

**TABLE 3:** Subgroup Analysis: technostress Mean (SD) by Gender, Job Type, and Education With Corresponding *T* Test (Two Groups) or One-Way Analysis of Variance (Three Groups)

	Overall technostress	Overload	Invasion	Complexity	Privacy	Inclusion
**Sex**						
Male	12.7 (2.1)	2.3 (0.8)	2.5 (0.8)	2.4 (0.7)	2.3 (0.7)	3.2 (0.7)
Female	13.3 (2.0)	2.3 (0.7)	2.8 (0.8)	2.5 (0.7)	2.3 (0.6)	3.4 (0.7)
	** *0.29*^a^		*** *0.39*^a^			* *0.27*^a^
**Job type**						
White collars	12.7 (2.0)	2.1 (0.6)	2.7 (0.8)	2.4 (0.6)	2.3 (0.7)	3.2 (0.6)
Blue collars	13.1 (2.1)	2.6 (0.8)	2.3 (0.8)	2.6 (0.7)	2.2 (0.6)	3.4 (0.7)
	* *0.22*^a^	*** *0.77*^a^	*** *0.41*^a^	** *0.32*^a^	* *0.20*^a^	** *0.30*^a^
**Education**						
≤Middle school	13.3 (2.1)	2.7 (0.8)	2.3 (0.7)	2.6 (0.7)	2.3 (0.7)	3.4 (0.6)
High school	12.6 (2.0)	2.2 (0.8)	2.5 (0.9)	2.4 (0.7)	2.2 (0.7)	3.3 (0.7)
≥Academic degree	12.8 (2.1)	2.0 (0.6)	2.8 (0.9)	2.4 (0.7)	2.3 (0.6)	3.2 (0.6)
	* *0.16*^a^	*** *0.35*^a^	*** *0.20*^a^			

*P < 0.05, **P < 0.01; ***P < 0.001.

^a^Effect size (Cohen’s d for two groups, Cohen’s f for three groups).

## DISCUSSION

This study examined the psychometric properties and validity of the revised technostress scale, originally developed by Nimrod, and here specifically tailored for older workers. Our results showed that this scale is a valid and reliable instrument, supporting its utility for assessing technostress among aging workers within different Italian working contexts. Consistent with previous research, we observed good psychometric properties attesting the scale’s reliability and validity.^13,30^ Compared to Nimrod’s study,^13^ we found similar average levels of technostress (13.8 vs 12.7), with the highest scores observed in the inclusion subscale, attesting that feelings of exclusion may be the most relevant source of technostress for older adults even in the workplace.

CFA revealed clear and distinct factors related to technostress and confirmed the original five-dimensional structure with acceptable fit indices, highlighting the scale’s ability to capture the multifaceted nature of this complex phenomenon among older workers.

Literature has largely shown the associations between technostress and negative psychological outcomes, including fatigue, irritability, anxiety, and emotional exhaustion.^7,8,31^ However, available research lacks findings among older workers as it mostly addressed the private life domain of older adults or work-related technostress in the general workforce, with few insights on age differences^6,32,33^; consequently, comparison between our findings and previous literature is limited.

Concurrent validity of Nimrod’s technostress scale was previously confirmed by its negative correlation with life satisfaction^13^; although different in psychological measures, our findings also showed a positive association between technostress and both impaired mental health and perceived stress, coherently to previous research.^31,34,35^

Research also found positive associations between ITC use and psychological well-being in older adults, suggesting the role of ITC in promoting healthy aging.^36,37^ Engagement with technology, including computers, smartphones, and other digital devices, is associated with enhanced cognitive functioning, increased social interaction and access to information and resources, overall contributing to improve mental health in later life. While the positive associations between technology use and psychological well-being in older adults are evident, it is essential to acknowledge potential challenges in the occupational context. Issues like digital literacy, access to technology, and concerns about privacy and security can affect how much older adults can take advantage of technology’s benefits. Tackling these challenges is essential for ensuring fair access and maximizing the positive impact of technology on the psychological well-being of the aging population.

In our study, we found technostress to be negatively correlated with workability (r = −0.24, *P* < 0.001). This finding is consistent with previous German research showing a negative association between digital work intensification and workability in a cohort study of older workers across different socioeconomic positions.^38^ Although studies evaluating technostress and workability among older workers are extremely rare, it might be assumed that technostress, similarly to other psychosocial risk factors, is associated with a decrease in workability.^39^

We also found an association between avoidant coping strategies and technostress (r = 0.20, *P* < 0.001), attesting the relationship among the use of maladaptive coping strategies and the negative impact of techno-stressors. Conversely, our results did not support the role of problem and emotion-focused coping strategies in mitigating technostress.^28^

Our analysis showed distinct technostress experiences between white-collar and blue-collar workers. Overall, white-collar workers reported lower levels of technostress compared to blue-collar. This difference could be attributed to the nature of tasks in each sector and their respective interactions with advanced technologies. Specifically, white-collar workers, often engaged in administrative roles, which depend on informatics systems, may experience concerns of invasion and privacy as technology increasingly permeates their work and personal spheres. Conversely, blue-collar workers, whose roles may involve direct interaction with evolving technologies like semiautomatic production and computer numerical controls (industry 4.0), experience stress in areas of overload, complexity and inclusion. This divergence underlined the interplay between technological advancement, occupational roles, and individual perceptions, highlighting the necessity for tailored interventions to address these challenges.

Similarly to previous research,^13^ we found females to report significantly higher technostress compared to men, particularly concerning the invasion and inclusion subscales. Literature on the relationship between gender and technostress showed divergent findings: Riedl^40^ and Ragu-Nathan^8^ found higher level of technostress for male subjects compared to women while other scholars showed women to be more prone to suffer from technostress than men, especially during COVID-19 pandemic.^35,41^ It has been noted that women may be at higher risk of experiencing techno-complexity and techno-uncertainty, whereas men can be more exposed to techno-overload and techno-invasion.^42^ Lack of clear results in this regard can be explained by the fact that studies have been conducted across various occupations and differences in job settings, role, education level and expertise which might influence research outcomes. Furthermore, none of these studies have focused on the older workforce, suggesting the necessity of comprehensive research of this demographic sector.

Our study has several limitations. First, our sample was drawn from specific occupational sectors, potentially limiting the generalizability of the findings to other contexts. Nonetheless, we included employees from three diverse companies with varied roles and responsibilities. Second, there was gender disparity with 76% of our sample being men, especially in blue-collar roles. This imbalance may impact the reliability of gender and worker type comparisons. For this reason, we calculated the effect size as a measure independent of sample size, to better assess the practical significance of the observed differences. Third, our sample was not completely heterogeneous in terms of age, with most participants aged 52 to 57 and few over 60, making the comparison among different age ranges difficult. Despite these limitations, our study is the first evaluation specifically targeted to workers psychological wellbeing in different occupational contexts, with a specific focus on older employees, as possible target population of technostress. Our findings suggest technostress to be a relevant issue for older workers’ wellbeing encouraging further research.

This validated technostress scale holds substantial implications for both research and practical interventions. As measures to prevent or mitigate technostress in the workplace are still scarce,^32^ this scale represents an accurate and reliable instrument to help organizations identify technostress levels among their aging workforce and implement targeted interventions. Furthermore, the findings encourage further exploration of specific factors contributing to technostress in this demographic, paving the way for tailored strategies to enhance workplace well-being. Addressing this issue and minimizing its effects begins with recognizing its impact on organizational performance, a positive technology perspective in the design of both technologies and work processes becomes the crucial first step in preventing technostress.^43^

Finally, our findings support the need to identify targeted interventions for older workers, which, according to literature, have yet to be fully determined.^18^ Future interventions could focus on enhancing digital literacy, improving access to training, and creating support systems to help older workers cope with technological challenges.

## CONCLUSIONS

Our study contributes to the literature on technostress by providing a thorough psychometric evaluation of a specialized scale among older workers in the Italian context. The scale’s demonstrated reliability, criterion and factorial validity, and associations with psychological and occupational outcomes underscoring its significance as a valuable tool for assessing and addressing technostress in the aging workforce. As organizations aim to create inclusive and supportive workplaces, this research offers practical insights into mitigating technostress among older employees, ultimately fostering a healthier, more productive and technology-oriented work environment.
